# Qualitative process evaluation of a problem-solving guided self-help manual for family carers of young people with first-episode psychosis

**DOI:** 10.1186/1471-244X-14-168

**Published:** 2014-06-06

**Authors:** Terence V McCann, Dan I Lubman

**Affiliations:** 1 Centre for Chronic Disease Prevention and Management, Discipline of Mental Health Nursing and Aged Care, College of Health and Biomedicine, Victoria University, PO Box 1428, Melbourne 8001, VIC, Australia; 2 Turning Point, Eastern Health and Monash University, Melbourne, Australia

**Keywords:** Bibliotherapy, Clinicians, First-episode psychosis, Guided self-help, Nurses, Primary carers, Problem-solving, Process evaluation, Qualitative research, Randomised controlled trial, Self-help manual

## Abstract

**Background:**

Caring for a young person experiencing first-episode psychosis is challenging and can affect carers’ well-being adversely. While some face-to-face approaches have achieved promising outcomes, they are costly and resource-intensive to provide, restricting their reach and penetration. Guided self-help in book-form (or bibliotherapy) is an alternative but untested approach in these circumstances. In this study, we aimed to evaluate carers’ beliefs about the usefulness of problem-solving guided self-help manual for primary carers of young people with first-episode psychosis.

**Methods:**

A qualitative process evaluation nested in a randomised controlled trial, conducted across two early intervention psychosis services in Melbourne, Australia. 124 carers were randomised to problem-solving guided self-help intervention or treatment as usual. We also undertook a qualitative process evaluation, using individual interviews, with a random sample of 24 of the intervention group. A thematic analysis of the qualitative data was undertaken, which is the subject of this paper. Interviews were conducted between January 2009 and September 2010.

**Results:**

Three themes were abstracted from the data, reflecting carers’ beliefs about the usefulness of the manual: promoting carers’ well-being, increasing carers’ understanding of and support for the young person with first-episode psychosis, and accessibility and delivery modes of the programme.

**Conclusion:**

This process evaluation highlights that guided self-help is useful in informing and supporting carers of affected young people. While there is scope for broadening the delivery modes, the approach is easy to use and accessible, and can be used as a cost-effective adjunct to standard support provided to carers, by community mental health nurses and other clinicians.

**Trial registration:**

ACTRN12609000064202

## Background

Caring for a young person with first-episode psychosis (FEP) is challenging and can lead to psychological distress [1], as well as adversely affect a carer’s experience of caring and their overall well-being [2]. It can also impact on their coping [3,4] and expressed emotion [5,6], with the latter associated with relapse and poor recovery outcomes for young person with FEP [7,8]. Yet most carers are ill-prepared to take on a caring role [2], with many feeling inadequately supported [9].

Although some FEP trials have incorporated family interventions as an element of a larger program of care, it has not been possible to determine their precise influence on carer outcomes [10]. However, many have been linked with favorable outcomes; for example, Gleeson *et al.*[11] conducted a randomised controlled trial (RCT) of young people with FEP and carers incorporating a wide-ranging intervention for families. Outcomes indicated caregiver associated stress improved significantly in the intervention group in comparison to the gold standard treatment as usual group [11]. Even though few other FEP family studies have been undertaken, a specialist FEP family program is regarded highly by carers and is linked with a significant reduction in carer stress [12]. However, notwithstanding their potential, such interventions are resource-intensive and expensive to implement, restricting their reach and dissemination, and are often problematic to access.

A different, less therapist-focused and cost-effective method of assisting carers is to use guided self-help in book-form (or bibliotherapy) [13,14]. Guided self-help can be undertaken by carers more or less independently of health care professionals [14,15]; though, the method appears to be more effective when combined with other therapeutic approaches [13]. It reduces travel to receive face-to-face therapy, is easily accessible and enables readers to re-read material subsequently [16-18]. However, most mental health based guided self-help studies have focused on individuals with anxiety and depression, not on carers. A meta-analysis of RCTs comparing guided self-help with face-to-face psychotherapy for depression and anxiety found comparable effects and no significant differences in drop-out rates between the two treatment modalities [14]. It is noteworthy that while the term ‘therapy’ is used mainly in the ‘treatment of patients with mental or psychological disorders by psychological means’ [19], it can also be applied to carers, who do not have these disorders, for a range of supportive measures such as education, training, problem-solving, and to enhance physical and/or psychological well-being.

The *Reaching Out: Supporting a Family Member or Friend with First-Episode Psychosis* manual is the first guided self-help study to focus on carers of young people with FEP. The manual is based on problem-solving therapy, ‘the self-directed cognitive-behavioural process by which a person attempts to identify or discover effective or adaptive solutions for specific problems encountered in everyday living’ [20, p.11]. The approach adopted in the present study involved facilitating primary caregivers to use the problem-solving process outlined by D’Zurilla and Nezru [20] to solve problems in their own lives and support their young family member with FEP.

A recent RCT of the *Reaching Out* manual [21,22] demonstrated that carer recipients of the problem-solving guided self-help intervention (PSGSHI) had a better experience of caring than recipients of treatment as usual (TAU), and these effects were maintained at both follow-up time points. The PSGSHI group had a better experience of caring for young people with FEP than the TAU group, and these outcomes were maintained at both follow-up time points. The PSGSHI group also experienced a greater reduction in negative emotional evaluations of the need to provide extra support to the young people with FEP than the TAU group by Week 6. Psychological distress decreased at a greater rate in the PSGSHI in comparison to the TAU group [22].

RCTs are commonly regarded as the ‘gold standard’ or the most effective way of evaluating interventions [23,24]. However, they are criticised frequently for being a ‘black box’ because they fail to explain why interventions work or fail [25]. This is attributable to their primary focus on assessing pre-specified outcomes and less so on the implementation process [26]. Process evaluation can fill this gap because it provides an in-depth understanding of how participants experience the intervention [27], as well as examining underlying facilitators and barriers to adopting the intervention [25]. The UK Medical Research Council [28] guidelines for developing and evaluating complex interventions, such as RCTs, recommend carrying out a process evaluation, in addition to an outcomes evaluation. Grant *et al.*[25] emphasise that process evaluation needs to be customised to the RCT, the intervention and outcomes being assessed. Most process evaluation research designs are qualitative [26,29] but can also be quantitative or mixed methods.

In this study, we undertook a qualitative process evaluation to evaluate carers’ beliefs about the usefulness of a problem-solving guided self-help manual for primary carers of young people with FEP.

*Definition*. A carer is the ‘main person (aside from health, social, or voluntary care provider) responsible for assisting with activities of daily living, supporting and advocating on behalf of the young person with FEP’ [2, p.382].

## Method

### Study design

A qualitative process evaluation was undertaken, using semi-structured, audio-recorded telephone interviews. Ethical approval was obtained from Melbourne Health and Southern Health research ethics committees. Written informed consent was obtained from participants, and confidentiality was maintained.

### Participants & procedure

Participants were first-time carers recruited through case managers of two specialist FEP centers, Orygen Youth Health and the Recovery and Prevention of Psychosis Service, both in Melbourne, Australia. Study inclusion criteria were: first-time carer (never previously been in a caregiver role), in the carer role for less than three years, and able to communicate in conversational English. Exclusion criteria were: had been a recipient of specialist family interventions for FEP, and recent personal history of serious and enduring mental illness.

124 carers were recruited and randomised to the PSGSHI (n = 61) and to TAU (n = 63). The majority were female (82.3%), a parent of the client (91.1%), resided with them (82.3%), and were born in Australia (65.3%). The majority of clients were in the recovery phase of their illness (85.3%). Carers indicated that their support role had affected their mental (76.4%) and physical (59.3%) health, socialisation (59.3%) and employment (62.7%) adversely [21,22].

TAU included specialist support, coordinated by a case manager and psychiatrist, with the family being engaged and integrated within the young person’s individual treatment plan. Printed basic information about FEP and additional support was provided, as required. The PSGSHI group received the *Reaching Out* manual, which contained an introduction and five modules, written in plain language that aimed to promote carers’ well-being and support them in their caregiving role (Table 1).

**Table 1:** Summary of Reaching Out guided self-help manual modules aims and content

**Module**	**Aims/Content**
**Introduction**	To (i) increase carers’ understanding of psychosis, and (ii) appreciate how to use the problem-solving framework.
	Content: Summary of psychosis; myths stereotypes and stigma about psychosis; the role of a carer; family and friends; common experiences of families; introduction to the 5-step problem-solving approach.
**1. Caregiver well-being**	To assist carers to: (i) work through their emotions, (ii) reflect upon how they are currently looking after themselves, and (iii) develop good coping techniques.
	Content: Carers’ emotions; wellbeing; coping skills and guidelines for coping; problem-solving activity.
**2. Getting the best out of support services**	To enable carers to (i) access support services, and (ii) get the most out of support services.
	Content: Carer’s rights and responsibilities; how to access and communicate with service providers; building relationships with service providers; a framework for asking questions from treatment providers; making complaints and voicing concerns; confidentiality; problem-solving activity.
**3. Well-being of the person with psychosis**	To (i) increase carers’ understanding of how they can promote the affected young family member’s well-being; (ii) equip carers to provide practical and emotional support to the young person; (iii) increase carer’s understanding of, and contribution to treatment; and (iv) recognise early signs of relapse.
	Content: Promoting the well-being of the young person with psychosis; emotional and practical support; how to prevent relapse; treatment; medication; stress management; family support; problem-solving activity.
**4. Dealing with the effects of the illness: Part 1**	To (i) examine effective ways of carers communicating with the young person; and (ii) equip carers about how to deal with the young person when they lack motivation, socially withdraw, engage in risky and unrestrained behaviour, have disturbed sleep, and experience hallucinations and delusions.
	Content: Effective communication; lack of motivation; social withdrawal; risky and unrestrained behaviour; disturbed sleep; hallucinations and delusions.
**5. Dealing with the effects of the illness: Part 2**	To explore ways for carers to respond to and support the young person when they are engaging in challenging and risky behaviour.
	Content: Weight gain; reluctance to take medication; substance misuse; aggressive behaviour; suicidal behaviour; depression and suicide; self-harm and suicide.

Carers worked independently through the modules over 5 weeks. The content of each module took up to two hours to complete and contained reading materials and exercises. Treatment adherence was assessed through weekly telephone calls from a research officer who asked the carer a set of standardised questions about the content of specific modules. The researchers who developed the manual were not involved in recruitment, weekly telephone calls or data collection.

### Data collection

Outcome assessments were conducted at baseline, 6- and 16-week follow-up. We also carried out a qualitative process evaluation (the focus of this paper) with a random sample of the PSGSHI group, who were recruited at the 16-week follow-up interview by the researchers. An interview guide was developed based on Lichstein, Riedel and Grieve’s [30] treatment implementation model for evaluating RCTs, and the content, format and delivery of the guided self-help manual (Table 2). The purpose of the interviews was to enable the researchers to ask a series of focused questions so carers could relate their beliefs about the usefulness of the *Reaching Out* manual. While the semi-structured interview approach guided the interview, it also allowed the researcher flexibility to explore answers [31]. Interviews lasted around 30 minutes, and no participants declined to participate or withdrew from the interviews.

**Table 2:** Sample of interview prompts relating to primary carers’ beliefs about the usefulness of the Reaching Out manual

1.	Which parts, if any, of the manual were most helpful to you in your role to provide support/care? Why were these parts of the manual most helpful?
2.	Which parts, if any, of the manual were least helpful to you in your role to provide support/care? How could these parts be improved in the manual?
3.	What difficulties, if any, did you have in completing the manual?
4.	What additional information, if any, should be included in the manual?
5.	What advantages, if any, are there in having a manual for providing information about how to give support/care?
6.	What disadvantages, if any, are there in having a manual for providing information about how to give support/care?
7.	Can you suggest other ways that could be used to provide information about how to give support/care?

### Data analysis

Braun and Clarke’s [32] six-step approach was used to analyse the data. (i) Transcripts were read and re-read to obtain a broad understanding of caregivers’ beliefs about the usefulness of the manual, and initial ideas were noted. (ii) Transcripts were examined closely and initial codes inserted. (iii) Codes were grouped into potential themes. (iv) Themes were reviewed and a thematic ‘map’ of the analysis was generated. (v) Themes were refined and grouped into themes and sub-themes. Saturation of themes with ‘thick’ description of the data was researched when no new data emerged to support each theme [33]. (vi) A more intense analytical arranging of themes and sub-themes, including selection of illustrative exemplars, occurred. Finally, Hill, Thompson & Williams’ [34] criteria were adopted to ascertain the representativeness of themes: ‘general’ — applies to all cases; ‘typical’— applies to half or more cases; ‘variant’ — applies to more than two but less than half the cases; and those that apply to only one or two cases are not reported.

A semantic level of analysis was undertaken, progressing from initial description and summary, in the results section, to interpretation, in the discussion section [32].

### Rigour

Sample size was established by saturation of themes with ‘thick’ description of the data [33,35], a key criterion of the rigour of qualitative methods in determining sample size [36,37]. Dependability and confirmability were maintained by briefing thoroughly the two researchers who collected data about using the interview guide, and by developing an audit trail to connect raw data and codes with themes and sub-themes. Initial coding and thematic analysis was undertaken out by TMcC, followed by an independent analysis by DL [38], and differences in themes were resolved through discussion. Credibility was enhanced by using a semi-structured interview guide to ensure a uniform method of interviewing [39]. Transferability was assured by presenting sufficient raw data here to allow readers to appraise the themes and to assess their transferability to other situations [33].

## Results

24 intervention group carers participated in the qualitative process evaluation; 22 females and 2 males; average age 47 years; a parent of, and living in the same household as, the client. Three general themes were abstracted from the data reflecting their beliefs about the usefulness of the *Reaching Out* manual: (i) promoting carers’ well-being, (ii) increasing carers’ understanding of and support for the young person with FEP, and (iii) accessibility and delivery modes of the programme.

### Promoting carers’ well-being

A central focus of the guided self-help programme was to promote carers’ general well-being. Two sub-themes were abstracted from the data relating to the usefulness of the manual in enhancing their well-being: equipping carers to maintain their own well-being and coping, and fostering identification with other carers.

#### Equipping carers to maintain their own well-being and coping

In this general sub-theme, the manual was helpful in placing emphasis on how carers could maintain their own well-being and coping. It helped to sustain them in their caregiving role by drawing their attention to their own needs and providing them with information about how to look after themselves: *“[It] teaches me how to deal with myself first. It was just getting to the point where I don’t know how to deal with things”.*

The manual was regarded as a good resource about carers’ coping mechanisms for stress related caring and for dealing with the young person’s behaviour. It helped them to cope better as carers: *“*[It’s a] *very good resource for coping mechanisms … it was a module on their* [the young people’s] *feelings with suicide, depression, how to cope with those. It helped me to be able to deal with my problem as well, my depression and that sort of thing”.* The problem solving tool, which was a central feature of the manual, helped carers cope with the young person’s behaviour: *“Coping with stress and the behavioural [aspects of the illness] is good; how to best manage stress and the constant interaction [with my daughter]. The problem solving tools helped me to cope with my daughter and not to put things at bay; not just to stand back”.*

#### Fostering identification with other carers

In this typical sub-theme, inclusion of realistic case studies in the manual gave carers an appreciation of the existence of others in a similar situation as themselves. This heightened awareness benefitted their well-being indirectly because they realised there were other people in a similar situation as themselves; they were not alone: *“Reading the manual makes me realise that there are more people similar to me; I’m not as isolated.”* This realisation provided them with a sense of belonging or identification with other carers in a similar situation as themselves: “[It] *brings about a common experience of carers, which gives belongingness”.*

### Increasing carers’ understanding of and support for the young person with FEP

The manual helped increase carers’ understanding of the young people’s experience of psychosis, and equipped them to support the latter. Two sub-themes were abstracted from the data reflecting this theme: enhancing understanding of how the illness affects the young person, and improving their ability to respond appropriately to the young person.

#### Enhancing understanding of how the illness affects the young person

In this general sub-theme, using the manual helped increase carers’ understanding of the ways young people experienced FEP. It enhanced their understanding of what the young people were going through, what to expect, and what they, as carers, could do to help the young person. In so doing, the manual provided helpful information and increased carers’ knowledge of FEP, such as symptomatology, signs of relapse, and effects of the illness on affected young people.

“Understanding the illness to begin with is helpful …, helps us to learn how to understand illness, listen better and support him. [The manual] was all helpful for someone with no prior knowledge of psychosis.”

*“*[The manual is] v*ery informative, easy to understand. I also found the signs and symptoms of relapse; my daughter was displaying it* [relapse] *and I didn’t know what it is.”*

#### Improving their ability to respond appropriately to the young person

In this general sub-theme, the carers commented that the manual went beyond giving factual information about FEP. It equipped them to problem-solve and provided them with a range of practical strategies, which together, helped them to respond appropriately to the young person: *“It gives me more alternatives and gives me insight into the problem”.* It placed emphasis on supporting the young people, listening to and being helpful to them: *“One of the helpful sides of the modules is just listening* [to the young person]*, listening better, pretty much. I had no knowledge about psychosis before”.* Carers commented favourably about how the manual increased their understanding of, and role in, treatment, including medications; particularly, in dealing with any reluctance to take medications. They also referred to the helpful way the manual addressed difficult issues such as increasing their sensitivity to depression and suicide in the young person, and how to recognise early warning signs of suicide and respond appropriately in these situations. Carers also found the manual helpful with other issues, such as dealing with lack of motivation and illicit drug use by the young person:

“Looking at options for problem-solving and trying again if it doesn’t work. Factors on motivation are really helpful; it reinforces the care for the carer and is helpful in making it a priority.”

“Every section was of equal help. The drug and alcohol was the big thing for us because that would be the part that we could relate to best.”

### Accessibility and delivery modes of the programme

Carers commented about the accessibility of the self-help manual approach in equipping them for their caregiving role, and about whether other modes of delivery should be used to provide the programme. Three sub-themes were abstracted from the data encompassing this focus: accessibility of the manual, providing choice in programme delivery modes, and accessing group support.

#### Accessibility of the manual

In this general sub-theme, carers commented positively about the manual being accessible from several perspectives. They regarded it as accessible because it was comprehensive, well-written, easy-to-read and understandable, and informative. Because of its nature and size, the manual was easy to access and portable and could be read in a variety of settings, such as at home, using public transport, and in waiting rooms. Another feature of its accessibility was carers could use it as a reference source at any time, in part or in its entirety, and in private. It was also convenient to read and overcame some of the difficulties carers faced in accessing standard FEP outpatient services, such as the inconvenience and time commitment in having to travel variable distances to attend appointments.

Absolutely, there is something that I could refer back to. If I am in a situation where I may be unsure of what to do and I don’t want to talk to anybody about it because sometimes I don’t want to get verbal feedback from anybody, I just pick up the book, have a read and assess your options from that rather than have a verbal discussion with somebody. It was easy because it had sub-titles and each title has a heading and after you’ve read it. It’s easy just to refer back to where it was.”

“Read the manual at your leisure, at your pace, and you can go back and look at it again.”

A few carers made suggestions about improving the manual. One recommended several blank pages should be added to the end to enable them to write down strategies they found helpful and unhelpful: *“[with] a DVD, you just watch it and put it away; with a manual you can write in it; a blank page to write chronological sequence like this works this time and that”.* Two others commented about the photographs in the manual; one proposed that more photographs should be included, while another felt the photographs should be less contrived and more realistic. *“I’m a well-educated person so the manual is good for me, more comprehensive, but there should be more positive photos. The case studies are very good”.* One carer commented that access to the information in the manual could be enhanced by the inclusion of an index at the end of the manual and a glossary, which would list and define key terms alphabetically: *“Maybe more on glossary or index”.*

#### Providing choice in programme delivery modes

In this variant sub-theme, some carers commented that more choice should be provided in the self-help programme’s delivery mode. They felt that it should be extended to include DVD and online formats. Two main benefits were identified in developing these alternative approaches. The first benefit is DVD and online approaches would make the programme more accessible, particularly to carers who are less motivated or able to read a manual. A DVD could be listened to and viewed more easily than a manual. Furthermore, these carers felt it was easier to incorporate additional pictures, graphics and other forms of delivery, such as audio-visual vignettes, in a DVD or online programme than a written manual: *“Online with more pictures and graphics. DVD is very good; it’s like something … watching TV”.* The second benefit in developing alternative modes of delivery, particularly DVD, is the potential inclusiveness of the approach. As such, it is much easier to include the whole family in viewing and discussing a DVD than a manual; therefore, a DVD increased the possibility of harnessing greater family involvement and support than a written manual: *“DVD would be good, it’s an attention grabber; others may be not good readers”.* A potential disadvantage of using a DVD only, highlighted earlier, is it lacked the accessibility and convenience of the manual and facility for the reader to actively write in it. Similarly, there was acknowledgement that an online programme was not suitable for everyone:

*“Not everyone is on-line savvy, may be* [better for] *younger people.”*

“DVD, audio would be good; online is not for me.”

In some circumstances, an online programme might be regarded as a possible deterrence because of a lack of familiarity with and access to computers and the Internet: *“On-line frightens me.”* Furthermore, not all carers have a computer as well as access to the Internet: *“Online is good, but not everyone has computers”.*

#### Accessing group support

In this variant sub-theme, carers had contrasting beliefs about the usefulness of small group discussion to supplement the self-help manual. Some commented that they should be given access to small group discussion: *“A group situation where speakers can come and speak [to carers]. I prefer engaging with people; a small group would be beneficial”.* Access to a small group would enable carers to engage with and share their experience and know-how with others in a similar situation. That access would be increased if it entailed minimal travel by carers: *“Small groups with a demographic of 15 minutes location”.* Moreover, group discussion could help carers overcome some of the isolation they experienced as carers:

“A small group could breakdown the modules and discuss them. I find it a lonely experience; having RAPPS is a blessing in disguise.”

“Shared experience with a small group definitely outweighs the reason for not being there.”

Group support was also seen as helpful, particularly when carers found it difficult to get in contact with FEP service staff: *“I phone … [name of FEP service omitted] and there is nobody available to talk to, and I don’t know what to do. I truly want to speak to someone and there’s nobody. I look at the number in the [telephone] book but they are not doing it now [providing the service I need] …. I don’t like talking to someone but I need to sometimes”.*

Several drawbacks were highlighted with small group support. There was some concern that participation in a group would increase carers’ vulnerability because they would be identified as a carer with a family member with FEP. There was unease too that group involvement might require unnecessary travel and would be less convenient than using the manual:

“A small group is too open, and others may not want to participate. The location and availability of participants are also a problem.”

“Small group is good but the location is a drawback for me.”

## Discussion

The aim of this study was to evaluate carers’ beliefs about the usefulness of a problem-solving guided self-help manual for primary carers of young people with FEP. The findings provide a strong endorsement of the helpfulness of the approach. The manual was perceived as being valuable in promoting carers’ well-being and coping, and enabled them to identify with other carers in a similar situation as themselves. The manual was also regarded as helpful in increasing carers’ understanding and support of the young person with FEP. It did this by enhancing their understanding of how the illness affected the young person and increasing their ability to respond appropriately to and support the young person. The manual was also perceived as being accessible, easy to read and understand, and some suggestions were made for enhancing its accessibility. Another consideration is several participants felt its usefulness could be increased if it could be combined with other delivery modes.

The findings of this process evaluation compliment the findings of our RCT, summarised earlier, that highlighted beneficial effects of the program [21,22]. The findings also accord with those of other studies that emphasise the importance of attention to carers’ own well-being [2,8] and the need for support from family and friends [40] and mental health professionals [41,42]. The value of the manual in increasing carers’ understanding of the way the illness affects the young person is also consistent with the findings of other studies [2,43] that underscore first-time carers’ uncertainty in this situation.

Regarding the accessibility of the manual, our study indicates there was strong support among carers to retain the book-form format of the self-help programme, with minor modifications. The book-form approach was accessible, convenient to use in a wide range of settings, could be re-read, and reduced travel, all of which accords with the findings of guided self-help research reported elsewhere [16-18]. At the same time, there was recognition that some carers might not be motivated to read a manual. It has been noted elsewhere that other limitations of guided self-help in book-form are that problems with visual acuity, and restricted reading and writing abilities, may decrease the usefulness of a self-help manual for some carers [17]. Furthermore, individuals whose first language is not English are likely to have lower literacy and numeracy skills than those whose first language is English [44], and this would have adverse implications for the use of form of guided self-help.

The findings of our study indicated that some participants felt a DVD might be preferable for some carers, especially those who might not be motivated or have difficulty in reading a manual. In addition, using a DVD would enable the family to watch the programme together and discuss caring related issues and, perhaps, contribute to collective sharing of caregiving responsibilities. Few rigorous studies have been carried out to evaluate the efficacy of DVDs for increasing carers’ well-being and support-giving skills, especially within the context of FEP. For instance, a feasibility study of the usefulness of DVDs and telephone-based coaching for carers of people with an eating disorder, by Sepulveda *et al.*[45], reported this method seems to be a satisfactory way of providing skill-based training and information for carers.

Another recommendation of some carer participants in our study is that an online version of the problem-solving guided self-help programme should be developed. While access to computers and high-speed internet is increasing and carers are becoming increasingly savvy in using the Internet, technical barriers and/or lack of familiarity with technology, particularly among older carers, could restrict accessibility to this intervention [46]. Moreover, the use of online interactive interventions for carers is very limited [47], more so for carers of young people with FEP (and schizophrenia in general). The few interventions that have been developed and evaluated have been primarily for carers of people with dementia [48,49]. For example, Beauchamp *et al.*[48] conducted a RCT of a worksite-based internet multimedia programme for employed family caregivers of people with dementia. The findings showed that programme recipients experienced significant improvements in several mental well-being outcomes, intention to seek help and perceptions of positive aspects of caregiving.

A further recommendation of some carer participants in our study is the manual could be combined with small group face-to-face discussion to provide more support and help overcome the isolation of being a carer. Other benefits of group discussion have been reported previously [11], including reductions in caregiver related stress. However, two potential barriers were raised about participation in face-to-face group discussion; difficulties in travelling to group meetings if they were held at an inconvenient location, and for a few participants, groups would increase their vulnerability by having to disclose they had a family member with mental illness. One potential way of overcoming geographical and vulnerability barriers is to include a range of online carer discussion forums, but this requires further study.

### Limitations

These results should be interpreted within the context of four limitations. First, while this qualitative process evaluation provides an in-depth understanding of carers’ beliefs about the usefulness of a guided self-help manual, generalisability is not achieved from sample representativeness but from the themes that are relevant to other carers and FEP guided self-help contexts [50]. Second, there are concerns that telephone interviews may result in a loss of visual cues [51,52]. However, this potential disadvantages is outweighed by the advantages of the approach [53]. Participants may be as relaxed on the telephone and willing to disclose personal information as face-to-face interviews [53]. Telephone interviews also provide rich data [54], enable greater geographical access to participants [54], decrease time and costs [55] and enable more participant anonymity [56]. Third, as a consequence of funding limitations, only participants with conversational English were included in the study; therefore, caregivers who were unable to meet this criterion were excluded. Finally, one aspect of the intervention that was not evaluated was the use of weekly telephone calls to assess treatment fidelity and to give carers the opportunity to ask questions.

## Conclusions

The findings of this process evaluation of the *Reaching Out* manual emphasise the important contribution of guided self-help in enhancing carers’ well-being and equipping them to support young people with FEP. While there is scope to make some improvements to the manual, and to extend the delivery modes to include DVD, online and small group discussion, the findings show it is easy to use and accessible, and is valued highly by carers. The findings have implications for policy, practice and research. From a policy perspective, the findings reinforce the need to address the needs of carers and better equip them to provide support to young people with FEP, through the use of guided self-help in book-form and other delivery modes such as online. From a practice stance, the findings make an important contribution to knowledge by highlighting the valuable role this approach can make as a cost-effective and readily accessible adjunct to standard support provided to carers, by community mental health nurses and other clinicians. From a research standpoint, the findings highlight the need to extend the reach of guided self-help in book-form to include carers whose primary language is not English, and to those living in remote locations and, in so doing, to undertake a rigorous evaluation of the effectiveness and usefulness of the approach to these carers.

## Competing interests

TMcC and DL declared no competing interest. In the past three years, DL has received speaking honoraria from Astra Zeneca and Janssen-Cilag, and has provided consultancy support to Lundbeck.

## Authors’ contributions

TMcC had a major role in the design and oversight of the study, carried out the data analysis, and had a major role in writing the paper. DL had a major role in the design of the study and in writing the paper. Both authors read and approved the final draft.

## Pre-publication history

The pre-publication history for this paper can be accessed here:

http://www.biomedcentral.com/1471-244X/14/168/prepub
